# Mental health research priorities in Australia: a consumer and carer agenda

**DOI:** 10.1186/s12961-018-0395-9

**Published:** 2018-12-12

**Authors:** Michelle A. Banfield, Alyssa R. Morse, Amelia Gulliver, Kathleen M. Griffiths

**Affiliations:** 1 Centre for Mental Health Research, The Australian National University, 63 Eggleston Rd, Acton, ACT 2601 Australia; 2 Research School of Psychology, The Australian National University, Acton, Australia

**Keywords:** Consumer and carer involvement, Mental health, Priority-setting, Research

## Abstract

**Background:**

The perspectives of mental health consumers and carers are increasingly recognised as important to the development and conduct of research. However, research directions are still most commonly developed without consumer and carer input. This project aimed to establish priorities for mental health research driven by the views of consumers and carers in Australia.

**Method:**

The project was conducted in two studies. Firstly, a face-to-face discussion forum held in the Australian Capital Territory (Study 1; *n* = 25), followed by a national online survey (Study 2; *n* = 70). Participants in both studies were members of the community who identified as a mental health consumer, carer or both. In Study 1, participants developed topics for mental health research in small group discussions, then voted on which topics, developed across all groups and sorted into thematic areas, were a priority. An online survey was developed from these research topics. Study 2 participants were asked to rate topics on a 5-point priority scale and rank the relative importance of the highest-rated topics.

**Results:**

At the forum, 79 topics were generated and grouped into 14 thematic areas. Votes on priorities were spread across a large number of topics, with the greatest overall support for research relating to integrating care that is sensitive to past experiences of trauma into mental health service delivery (trauma-informed care). Survey responses were similarly spread, with the majority of research topics rated as important by at least 50% of participants and no clear individual priorities for research identified. Amongst items rated as important by approximately 80% of participants, key research areas included the delivery of services, and consumer and carer involvement.

**Conclusions:**

Australian mental health consumers and carers demonstrate a strong understanding of the mental health system and its inadequacies. Although clear specific priorities are difficult to establish, consistent areas of focus are services and the role consumers and carers can play in their improvement. However, for consumer and carer views to be at the forefront of research, it is important to regularly update research agendas and work in partnership across the whole research process.

**Electronic supplementary material:**

The online version of this article (10.1186/s12961-018-0395-9) contains supplementary material, which is available to authorized users.

## Introduction

The importance of consumer and carer involvement in mental health research is well established [1–8]. In Australia, the Statement on Consumer and Community Participation in Health and Medical Research outlined the importance of consumers and the community playing an active role in health and medical research [9]. Growing acceptance of the mental health recovery movement creates a solid foundation for consumers and carers to move beyond tokenistic or advisory modes of participation and into meaningful and effective involvement [10].

Internationally, these efforts have increased the consumer and carer voice in priority-setting [7]. For example, the James Lind Alliance initiative (based in the United Kingdom) and the Roadmap for Mental Health Research in Europe project have included consumers, carers and other stakeholders in large-scale priority-setting exercises for research into specific disorders and mental health more generally [11, 12]. However, published examples of consumers and carers defining the research agenda and actively participating in research in Australia are limited [10]. Although the research community uses a number of priority-setting methods, consumer and carer perspectives remain largely absent in mental health research priority-setting [3]. Given their unique and valuable perspectives in identifying areas and issues of emerging importance [3], or those that have otherwise been overlooked [2], consumers and carers have much to contribute with significant capacity for leadership in this space [13].

ACACIA – The Australian Capital Territory (ACT) Consumer and Carer Mental Health Research Unit – at The Australian National University (ANU), was established in response to the need for consumer- and carer-led collaborative research. ACACIA aims to enable consumers and carers to take an active role in relevant, high quality mental health research [14]. The Unit is led and staffed by researchers with lived experience as a consumer or carer who facilitate the engagement of consumers and carers from the community in the work of the Unit, and bridge the often difficult gap between academia and mental health communities [6].

A key objective of ACACIA is the collaborative development of a research agenda to address consumer- and carer-identified issues, such as service gaps in the ACT and Australia more broadly. This paper reports the findings from two consecutive studies to address this objective, namely a face-to-face discussion forum to develop topics and set initial priorities (Study 1) and an online survey to extend and update priorities (Study 2).

The ethical aspects of the research were approved by the ANU Human Research Ethics Committee (protocol number 2013/388). All participants provided written or online informed consent.

## Study 1: discussion forum

### Method

#### Participants

Participants who self-identified as consumers and/or carers were invited to participate, recruited via advertisements distributed through the mailing lists of ACT consumer and carer networks. Advertisements were also emailed to ACT members of the Depression and Anxiety Consumer Research Unit Register, a database of people who have expressed an interest in research conducted at the ANU Centre for Mental Health Research (CMHR).

#### Procedure

A face-to-face discussion forum was held in November 2013. Consistent with ACACIA’s commitment to consumer and carer leadership, all those involved in running the forum had lived experience of mental health issues. A local well-known consumer advocate facilitated overall proceedings, and small group discussions were facilitated by ACACIA staff (*n* = 2) or ACACIA Consumer and Carer Advisory Group members (*n* = 2). Small groups of 6–8 participants selected their own seats at one of four tables, each of which had a note-taker and a facilitator present. Facilitators assisted groups to express and formulate their ideas into topic areas that could be researched.

The detailed protocol for the discussion forum is provided in Additional file 1. The forum priority-setting comprised three parts, as follows: (1) identifying broad research areas within discussion groups, (2) refining the ideas into specific topics and questions, enabling participants to participate in the early stages of a modified thematic analysis [15], and (3) prioritising the research topics. Researchers and Advisory Group members collated the topics and questions into broad thematic areas by consensus, using an inductive approach [16]; themes were displayed on flipcharts in the forum venue for the prioritisation. Priorities were identified using a “*dot-mocracy*” [17] process. Each participant was provided with five coloured adhesive dots. The colour of the dot indicated which group – consumer, carer, or people who identified as both (hereafter called consumer/carers) – the participant self-identified with. Each dot represented one vote for a topic or thematic area. Participants were free to distribute their dots across as many or few topics as they wished.

#### Analysis

As described above, inductive thematic analyses [15, 16] were an integral part of the forum proceedings. Thematic analysis methods [13] were adapted to facilitate the development of topics for future research. Instead of developing descriptive codes, forum participants developed and refined research topics from the content of their discussions. These topics were collated and assigned to themes by the consumer and carer researchers, assisted by Advisory Group members. Results comprise the number of votes each topic or thematic area received from consumers, carers and consumer/carers. Further thematic analyses were conducted by researchers after the forum to produce a final list of research themes. The findings from the forum were circulated to participants for feedback. No further refinements were made.

### Results

#### Participants

A total of 25 people (17 female, 8 male) with lived experience as a consumer and/or carer attended the forum. Of these participants, 14 who identified as consumers, five as carers and five consumer/carers participated in the priority-setting exercise (one consumer participant left before the exercise was conducted). No further demographic information was collected from forum participants.

#### Research priorities

Seventy-nine topics for research in 14 broad thematic areas were developed by participants and collated for prioritisation at the forum. The themes were services; treatment; medication; health professionals; comorbidity and physical health; justice; consumer and carer involvement; stigma; experiences of care; carers, family and friends; National Disability Insurance Scheme (NDIS); language and communication; peer to peer; and legislation. Additionally, a list of individual ‘other’ topics (e.g. recovery and fulfilling potential, employment) was compiled. The available 120 votes (70 from consumers, 25 from carers and 25 from consumer/carers) were broadly distributed across 59 of the 79 topics (Additional file 2).

Research on trauma and service delivery was highly rated: ‘the integration of trauma-informed care into service delivery’ was considered important by all three participant groups and received the highest overall number of votes (*n* = 7), and ‘is care traumatising’, received 5 votes (3 from consumers and 2 from carers). ‘Peer-led services’ and ‘recovery and fulfilling potential’ were also considered important, each receiving six votes.

There were some differences in the focus of research priorities between consumers, carers and consumer/carers. Whilst consumer/carer votes were distributed widely across topics, the votes for research into ‘peer-led services’ and ‘recovery and fulfilling potential’ were primarily from consumers, and no participants identifying solely as a carer voted for these topics. Consumers were the only group to vote for research into the effects of ‘stigma’, ‘human rights legislation’ and ‘learned helplessness in response to experience of services’. Carers focused on research related to ‘interaction with health professionals’ and the ‘care experience’, including the influence of ‘privacy’ on care, as well as the effects of ‘drug and alcohol use’.

## Study 2: priority-setting survey

### Method

To update the research agenda developed in 2013 and extend participation nationally, a second study of research priority-setting, comprising an online survey, was undertaken in 2017. The methods used were based on a previous survey investigating priorities for depression and bipolar disorder research conducted by the lead author [2].

#### Participants

To recruit participants for the online survey, advertisement targets used for the discussion forum were expanded to include both national and state-based health consumer and carer organisations. Advertisements were also disseminated through the Lived Experience Research register (a database of consumers and carers who have expressed interest in participating in ACACIA research), the CMHR website and the CMHR social media accounts. Recruitment flyers were distributed at community events during National Mental Health Week. Survey participants were required to be at least 18 years old and live in Australia.

#### Procedure

The survey was conducted over 8 weeks in September and October 2017. Survey items were developed from the research topics formulated by participants at the forum, specifically taking care to preserve original wording. Additional items that were not well-represented by the original topics presented to forum participants were developed from the detailed notes on the small group discussions in Study 1. Eighty-seven items were included in the survey, each representing a single topic. Items were presented within the 14 broad thematic areas that were identified by researchers at the forum. Demographic information (age, sex, identification as a consumer and/or carer, state/territory of residence) was collected at the beginning of the survey. A copy of the survey is provided in Additional file 3.

Survey participants were asked to rate the priority of each research topic on a 5-point Likert scale (1 = Very low priority to 5 = Very high priority). All items an individual participant rated as of very high priority (5) were collated by the survey software. Participants were then asked to rate all items they designated as high priority in order of relative priority using a ‘drag and drop’ process. To encourage variation in an individual’s ratings and create a manageable list to rank, participants were informed about the ranking process before commencing the survey, and reminded at the top of each page that anything rated as ‘very high priority’ would appear in the ranking list. However, feedback provided in the comments section indicated that the ranking process proved too difficult for most participants, raising serious concerns about the validity of the relative rankings. Results are therefore restricted to analysis of the ratings data as described in the next section.

Participants were also invited to suggest new research topics or provide comments in open-ended questions at the end of each theme and after the ranking exercise.

#### Analysis

##### Ratings

Due to the large number of survey items and relatively low number of participants, inferential statistics were not used to compare ratings between groups. A descriptive analysis was performed, examining the distribution of ratings and comparing the content of the highest-rated items. All quantitative analyses were performed in IBM SPSS Statistics 25.

Missing data for each item ranged from 1.4% to 20% and progressively increased across the survey. The order of survey items was not randomised and items presented later in the survey appear to have been impacted by participant fatigue. Due to the pattern of missing data, participants were included in descriptive analyses if they had completed any of the rating items. No participants or variables were excluded from the analysis based on missingness.

The distribution of responses to individual rating items were visually examined and a majority of items were found to be negatively skewed. Based on this observation, participant ratings were dichotomised [18] as follows: important (combining very high (5) and high (4) priority ratings) versus all other (combining moderate (3), low (2) and very low (1) priority ratings). For each participant group (consumers, carers and consumer/carers), the percentage of participants whose ratings were in the ‘important’ category for each item was calculated. To address missingness and drop-out across the survey, a valid percentage was calculated based on the number of participants who completed an item, not the total number of participants. Items were then ranked within participant groups in descending order of the percentage of ‘important’ ratings. Tied rankings were assigned the mean rank [19]. The distribution of ‘important’ ratings across items and groups was examined for consensus on clear ‘top’ priorities or cut-off points for the highest priorities for research.

##### Open-ended responses

Qualitative analyses of the survey comment data were conducted by one author (ARM) and managed using QSR International’s NVivo 11 Software. Participants’ open-ended responses were examined using a framework analysis approach [20]. The coding framework was developed from the key areas for research identified in the descriptive quantitative analysis. Carer participants provided written responses more frequently than consumer and consumer/carer participants; thus, the framework analysis findings may favour carer priorities and concerns. Qualitative findings are incorporated with quantitative findings to provide additional detail.

### Results

#### Participants

Table 1 presents the demographic data for survey participants. A total of 70 consumers and/or carers participated in the online survey, including 37 consumers, 12 carers and 21 consumer/carers. The mean age of participants was 46.3 years (SD 15.8, range 21–83). All Australian states and territories were represented in the sample, although the majority of participants were from the ACT, New South Wales and Victoria.

**Table 1:** Survey demographic data

	Number	Percent
Group		
Consumer	37	52.9
Carer	12	17.1
Consumer and Carer	21	30.0
Total *N*	70	100
Gender		
Male	14	20.0
Female	52	74.3
Other^a^	3	4.3
Prefer not to say	1	1.4
Total *N*	70	100
State or Territory		
Australian Capital Territory	26	38.2
New South Wales	16	23.5
Northern Territory	2	2.9
Queensland	2	2.9
South Australia	2	2.9
Tasmania	1	1.5
Victoria	14	20.6
Western Australia	5	7.4
Total *N*	68	100

^a^Non-binary trans-masculine = 1, genderqueer/genderfluid = 1, not specified = 1

#### Research priorities

Table 2 presents the percentage of ‘important’ ratings for each item by participant group. Items were ranked within participant groups based on relative percentage (i.e. 1 = highest percentage of ‘important’ ratings; 87 = lowest percentage). No consensus on clear ‘top’ priorities for research was observed. Most items were rated as important by at least 50% of consumers (81 items), carers (60 items) and consumer/carers (68 items). A small number of items were rated as important by all, or almost all, participants within a group. However, the percentage of ‘important’ ratings gradually decreased across items (Table 2) with no obvious cut-off point for top research priorities.

**Table 2:** Research priority ratings for consumers, carers and consumer/carers

Research topic	Topic area	Priority ranking (percentage important ratings)		
		Consumer	Carer	Consumer/Carer
How to implement internationally recognised models of peer support in Australia	*Peer to Peer*	1 (94)	24 (70)	72.5 (44)
Over-representation of mental illness in the justice system	*Justice*	2 (88)	1.5 (100)	11 (78)
How is psychosocial disability defined in the NDIS, and how will it impact consumers and carers in Australia?	*National Disability Insurance Scheme*	4.5 (88)	9 (80)	15.5 (76)
How does the use of language include/exclude individuals?	*Language and Communication*	4.5 (88)	69 (44)	55.5 (56)
Consumers’ experiences of peer-to-peer services	*Peer to Peer*	4.5 (88)	39 (60)	64.5 (50)
Social inclusion	*Other*	4.5 (88)	15.5 (78)	44.5 (60)
How participation works in practice (tokenism vs. real involvement)	*Consumer and Carer Involvement*	7.5 (85)	24 (70)	11 (78)
What is helpful in recovery-oriented services?	*Experiences of Care*	7.5 (85)	9 (80)	32 (65)
Peer-led services – What are the gaps? (e.g. support groups)	*Peer to Peer*	10 (84)	46.5 (56)	55.5 (56)
How to recruit and train peer workers – What is going on, and where? Where is it embedded? How are they being supported?	*Peer to Peer*	10 (84)	39 (60)	55.5 (56)
Mental health in LGBTIQ+ populations	*Other*	10 (84)	46.5 (56)	38.5 (63)
How is the consumer and carer voice integrated into policy and services? How are their contributions valued, and what indicators exist to demonstrate how their voice is used?	*Services*	12 (84)	52.5 (55)	21.5 (71)
How do we expand who is involved? (e.g. young people)	*Consumer and Carer Involvement*	13 (82)	15.5 (78)	64.5 (50)
Is medication what we want? Side effects, health impacts, alternatives, efficacy, cost-effectiveness	*Medication*	14 (79)	34 (64)	59 (55)
Stigma by health providers (mental health and others) – What do they believe and how does it impact?	*Stigma*	15.5 (79)	24 (70)	5 (83)
Are consumers being consulted about their experiences of care?	*Experiences of Care*	15.5 (79)	24 (70)	21.5 (71)
Impact of service delivery on consumers and carers – What contributes to recovery?	*Services*	17 (78)	58 (50)	7 (81)
What programmes/supports can be devised for reaching individuals that are outside of NDIS scope?	*National Disability Insurance Scheme*	20.5 (78)	9 (80)	15.5 (76)
Peer support in public mental health system	*Peer to Peer*	20.5 (78)	46.5 (56)	24.5 (69)
Accommodation	*Other*	20.5 (78)	46.5 (56)	25.5 (69)
Employment	*Other*	20.5 (78)	5 (89)	7 (81)
Support in education settings	*Other*	20.5 (78)	15.5 (78)	77 (38)
Culturally and linguistically diverse perspectives within mainstream mental health system	*Other*	20.5 (78)	69 (44)	32 (65)
How do individuals adapt to changes in medication that impact lifestyle and quality of life?	*Medication*	24 (77)	52.5 (55)	38.5 (63)
Training of psychologists – How can consumer perspectives be incorporated?	*Health Professionals*	26.5 (76)	62.5 (45)	44 .5 (60)
Where do physical health concerns fit into health services when you have mental health problems as a main focus?	*Comorbidity and Physical Health*	26.5 (76)	39 (60)	11 (78)
Discrimination	*Justice*	26.5 (76)	15.5 (78)	27.5 (67)
How have people who have experienced trauma been cared for?	*Experiences of Care*	26.5 (76)	39 (60)	21.5 (71)
Trauma informed care – Why is it important, and how is it integrated into service delivery?	*Services*	29.5 (76)	39 (60)	42 (62)
Reach – Are services reaching the people that need them?	*Services*	29.5 (76)	3 (91)	27.5 (67)
Capacity for decision-making/change in legislation and its application; consumer and carer experiences of this. What information is provided about legislation? What support is provided, e.g. legal?	*Legislation*	32.5 (75)	15.5 (78)	47.5 (59)
To what extent do we follow human rights legislation on mental illness?	*Legislation*	32.5 (75)	24 (70)	15.5 (76)
Recovery and fulfilling potential	*Other*	32.5 (75)	15.5 (78)	64.5 (50)
Suicide: continuous care and support	*Other*	32.5 (75)	5 (89)	7 (81)
Is care traumatising?	*Experiences of Care*	35 (74)	9 (80)	15.5 (76)
Consumer and carer journey through service pathways – What works and what doesn’t? What do clinicians think?	*Services*	36.5 (73)	34 (64)	52 (57)
Care coordination between mental health and physical health	*Comorbidity and Physical Health*	36.5 (73)	58 (50)	1 (89)
Consumer perspectives on use of labels – Which terms are useful/helpful, which are not?	*Language and Communication*	38.5 (72)	69 (44)	47.5 (59)
Mental health in culturally and linguistically diverse populations	*Other*	38.5 (72)	58 (50)	32 (65)
Children of people with mental illness	*Other*	40 (71)	29.5 (67)	38.5 (63)
Awareness and role of GPs, e.g. engagement with carers, language and communication skills with consumers and carers	*Services*	41.5 (70)	62.5 (46)	15.5 (76)
Exhaustion and burnout of mental health professionals – Impact on service support and delivery	*Health Professionals*	41.5 (70)	34 (64)	32 (65)
How many people with mental illness/disability are eligible for NDIS support?	*National Disability Insurance Scheme*	44.5 (69)	46.5 (56)	60 (53)
What forms of communication work for consumers and carers? (e.g. older people – less technology familiarity; younger people – social media, smart phones)	*Language and Communication*	44.5 (69)	84 (30)	19 (75)
Care planning – What makes a good mental health plan? (e.g. individualised, including perspectives of consumers, carers and clinicians)	*Treatment*	44.5 (69)	34 (64)	3.5 (84)
Transparency of clinical management – How does it respond and interact with consumers and carers?	*Treatment*	44.5 (69)	58 (50)	3.5 (84)
What are the experiences of and needs of people coming off medication? How are they being supported?	*Medication*	47 (68)	62.5 (45)	9 (80)
How mental health-aware are GPs?	*Health Professionals*	49.5 (67)	34 (64)	38.5 (63)
What is the role of a GP (perceived and actual) as part of the therapeutic alliance in care of mental health consumers?	*Health Professionals*	49.5 (67)	52.5 (55)	26 (68)
Stigma around borderline personality disorder	*Stigma*	49.5 (67)	81 (33)	76 (39)
Stigma as a barrier to consumer involvement	*Stigma*	49.5 (67)	46.5 (56)	43 (61)
Alternative treatments – What are they, and how can they contribute to recovery? Holistic approaches, meditation, exercise	*Treatment*	52.5 (66)	76.5 (36)	75 (40)
Learned helplessness (experience with services)	*Other*	52.5 (66)	69 (44)	55.5 (56)
Consumers and carers – Who is involved?	*Consumer and Carer Involvement*	55 (64)	15.5 (78)	55.5 (56)
Does the stigma in the mental health system worsen outcomes?	*Stigma*	55 (64)	29.5 (67)	32 (65)
What is the effect of caring?	*Carers, Family and Friends*	55 (64)	5 (89)	15.5 (76)
What sources of information do consumers and carers have faith in?	*Language and Communication*	57 (63)	81 (33)	64.5 (50)
How can medications be tailored to the individual?	*Medication*	58.5 (62)	62.5 (45)	50.5 (58)
Criteria for prescribing medications	*Medication*	58.5 (62)	52.5 (55)	38.5 (63)
What are clinician views on peer support?	*Peer to Peer*	60.5 (61)	81 (33)	72.5 (44)
Risk factors for mental illness	*Other*	60.5 (61)	46.5 (56)	32 (65)
How can we get mental health and other health professionals to work together more efficiently?	*Health Professionals*	63.5 (61)	20 (73)	50.5 (58)
Comorbidities and stigma	*Stigma*	63.5 (61)	69 (44)	64.5 (50)
What changes do people make in their own lives as a result of stigma?	*Stigma*	63.5 (61)	46.5 (56)	55.5 (56)
Who are the carers and what are they doing?	*Carers, Family and Friends*	63.5 (61)	24 (70)	47.5 (59)
Translation of clinical frameworks and guidelines into practice – Why is there a disconnect?	*Treatment*	66 (60)	52.5 (55)	38.5 (63)
Carers and bereavement – Are we offering enough counselling? Is it timely enough? Should it be offered in prisons?	*Carers, Family and Friends*	67.5 (59)	9 (80)	32 (65)
What is a peer?	*Peer to Peer*	67.5 (59)	81 (33)	86 (19)
What support is available when pain is a comorbid condition? How are people experiencing that?	*Comorbidity and Physical Health*	70.5 (58)	85 (22)	64.5 (50)
What is the evidence base linking mental illness with alcohol and other drugs?	*Comorbidity and Physical Health*	70.5 (58)	29.5 (67)	64.5 (50)
What kind of support would carers like?	*Carers, Family and Friends*	70.5 (58)	1.5 (100)	21.5 (71)
Is there such a thing as carer recovery?	*Carers, Family and Friends*	70.5 (58)	15.5 (78)	2 (88)
How do current protocols support consumer and carer journeys to recovery?	*Treatment*	73.5 (57)	24 (70)	69 (47)
How are Partners in Recovery, Personal Helpers and Mentors, support and clinical management working together?	*Services*	73.5 (57)	76.5 (36)	74 (43)
Bullying	*Other*	75 (56)	81 (33)	72.5 (44)
Trial of primary healthcare nurse within mental health teams – Does it improve physical health outcomes?	*Comorbidity and Physical Health*	76.5 (55)	74 (40)	84 (28)
Analysis of stigma according to disorder	*Stigma*	76.5 (55)	69 (44)	47.5 (59)
Bereavement	*Other*	78 (53)	69 (44)	82 (31)
How is ‘privacy’ interpreted by health professionals, and does it differ from consumer and carer interpretations?	*Health Professionals*	79.5 (52)	52.5 (55)	81 (32)
Stereotype formation	*Stigma*	79.5 (52)	58 (50)	79.5 (33)
Insurance	*Other*	81 (50)	29.5 (67)	85 (25)
Gender-specific effects of medication	*Medication*	82 (47)	87 (18)	78 (35)
Effects of drug and alcohol use early in life	*Comorbidity and Physical Health*	83 (39)	69 (44)	72.5 (44)
Electroconvulsive therapy – What information is given, does it follow best practice, what are consumers’ experiences?	*Treatment*	84.5 (31)	86 (20)	83 (30)
Smoking cessation	*Other*	85.5 (31)	69 (44)	87 (7)
Do the public and private sectors work together? Consumer and carer experiences	*Services*	86 (30)	76.5 (36)	79.5 (33)
Pet therapy	*Treatment*	87 (26)	76.5 (36)	64.5 (50)

Note: Items are ranked in descending order of consumer importance rating percentages (from 1 (most frequently rated as ‘important’) to 87 (least frequently rated as ‘important’)). The percentage rankings for the carer and consumer/carer participant groups are provided for comparison. Items sharing the same importance rating percentage were assigned a mean rank [19]

*GP* General Practitioners, *LGBTIQ+* Lesbian, Gay, Bisexual, Transgender, Intersex or Queer, *NDIS* National Disability Insurance Scheme

Participants’ open-ended responses further emphasised the ongoing need for research and evaluation across a broad range of topics, reflecting on the extent of perceived problems with the system.

“*I hate to say this but the list* [of research priorities] *touches directly on most of our carers and consumers involved in the mental health system. Your list shows just how far we have to go to have a first class mental health system*…” (Carer 1)

In the absence of clear top priorities, to develop initial key areas for future research, a pragmatic decision was taken to examine items rated as important by approximately 80% or more of participants within a group. Across groups, this demonstrated a focus on research topics related to the delivery of services. Participants rated topics about the quality of services, problems with services (including reach, stigma and trauma) and how services impact on consumers and carers as priority areas for future research. The NDIS (ndis.gov.au) was rated as an important topic for all groups. The NDIS is a national Australian programme that was trialled in a number of local sites from 2013, and began a national implementation process in 2016. The programme provides government-funded flexible packages of care to Australians with permanent and significant disability, including psychosocial disability. NDIS-related research topics focused on the impact of the ‘definition of psychosocial disability’ and ‘supports for people who fall outside the scope’ of the scheme.

Open-ended responses also had a substantial focus on the problems with services. Participants shared concerns about under-resourced, under-staffed services that could be difficult to access, poorly coordinated and poorly implemented.

“*Where I live … it is really difficult to get a care plan in either the hospital or community setting as the staff do not see the value and feel like they haven't got enough time to do things including writing and reading case notes.*” (Consumer/Carer 1)

Concerns were also raised about the mental health training of health professionals, particularly General Practitioners, whether the current approaches of health professionals to diagnosis and care were appropriate, and gaps in services, including gaps created by the implementation of the NDIS.

Alongside the focus on problems with services, some participants also highlighted the negative impacts of services on consumers and carers. Participants commented on the negative consequences of compulsory treatment and inadequate, inappropriate or absent services.

“*I was also treated in quite a paternalistic and demeaning way by* [the crisis response] *team, hospital staff and other staff around these issues as I was not a family member. The feeling of seeing someone you care for being treated in a completely degrading manner but also realising that no one cares about your voice is very horrible.*” (Carer 2)

Consumer and carer involvement was also a priority for research, with interest in ‘how involvement works in practice’ and ‘how to expand who is involved’ rated highly, particularly by consumers. Comments highlighted the role of active consumer and carer involvement in improving the quality of services and the education of mental health professionals (including General Practitioners and psychologists). Participants were interested in determining how to encourage diversity in the consumers and carers involved in shaping services, policy and legislation.

“*Lived experience will really matter to shaping better services, and making sure it's a good cross section of people from various socio-economic backgrounds, genders, ethnicity etc.*” (Carer 2)

Other topics frequently rated as important across the three groups included the ‘over-representation of mental illness in the justice system’, ‘social inclusion’ and ‘employment’. A small number of participants commented on these topics, emphasising the importance of ensuring people have access to basic needs, including accommodation, vocational activities and social support services, and that they received appropriate mental health care if detained in the justice system.

Despite an overall focus on delivery of services, there were some group differences on the specific topics of interest. Consumers frequently rated the implementation and consumer experiences of ‘peer services’ as important topics for research, with four of their top topics on peer-to-peer services. Carers most frequently prioritised ‘carer support services’, the ‘reach’ of services and ‘continuous care and support for suicide’. By contrast, consumer/carer participants focused on the organisation of care, including ‘care coordination’, ‘care planning’ and ‘transparency of services’. They also prioritised ‘carer recovery’.

## Discussion

The current study identified a broad range of topics for future mental health research, reflecting the extent of the perceived gaps in the Australian mental health sector. Although there were some group differences and no clear priorities emerged in either research study, many topics amongst the highest-rated were in the area of service organisation and delivery, particularly related to trauma- and recovery-oriented care and peer leadership. The importance of lived experience of mental health issues (as a consumer and/or carer) for the development and evaluation of services and policy was also a focus, particularly for survey participants, and across both studies there was interest in research into recovery for consumers and carers.

This research both confirms and extends the outcomes of previous consumer and carer mental health research priority-setting exercises. Many research priorities developed by participants in Study 1, such as support for transitions between services, medication and alternative treatments [7, 21], stigma [8, 21], support for carers [21], and communication with health professionals [21] are consistent with prior research conducted in other countries. The topic areas of focus, including recovery [21], service delivery [21] and the active involvement of consumers have also been found previously [8]. The findings are similarly consistent with previous Australian research priority-setting work, in which consumers prioritised research into medication and treatment, effective coping strategies/recovery and quality services [2, 3]. However, the topics developed and prioritised across both studies in the current project reflect specific contemporary issues in these areas. For example, many participants were aware of international evidence for mental health peer work and the spread of peer services in Australia; therefore, their suggested topics were focused on implementation and evaluation of best practice in the Australian context, including peer leadership in service delivery. The strong interest by participants in the effect of the recently introduced NDIS, particularly regarding how it affects service availability for those ineligible for the scheme, is also a critical area of concern in the Australian system, although the system-level analyses of interest may also have relevance for other countries in Europe and North America that have introduced similar broad-ranging changes to the financing and organisation of care for people with long-term disabilities [22].

The contemporary and nuanced views on research priorities demonstrated by consumers and carers, along with the changes in emphasis that occurred in the 4 years between studies, highlight the importance of regular updates to priorities that are not confined to rating pre-existing researcher-developed topics. For example, in Study 1 of the current research, topics focused on trauma were discussed widely across groups and rated highly by both consumers and carers, albeit by a small number of votes overall. Trauma-informed care refers to mental health care which acknowledges the impact of previous psychosocial violence, abuse and trauma on the consumer and is sensitive to these experiences [23]. However, trauma-related service delivery was not amongst the highest priority topics in the national survey conducted four years after the first study. This may reflect differences in focus across communities. For example, there may have been a pre-existing emphasis on trauma-informed care within the local ACT consumer network from which participants were recruited for Study 1. Alternatively, this may reflect a recent broader shift in the focus of both service delivery and the consumer movement given that the impact of previous trauma on mental ill health has become increasingly well recognised over time [24].

Although participants across both studies embraced the opportunity to develop research topics, the majority of topics were negatively skewed as participants rated most topics as ‘high priority’ overall. Participant comments in both studies suggested that this reflected the view that everything was equally important. They found it challenging to prioritise some topics over others, commenting that it was difficult to choose between numerous competing priorities. However, it is also possible that a different choice of response style (e.g. forced choice of a limited number; consensus method) may have produced different results. These methods were discussed, but considered less desirable by the ACACIA Advisory Group. Difficulty selecting priorities is an observation also reported in several previous research priority-setting projects [2, 3, 21]. Like the present project, these projects all enabled participants to independently select, rank or vote for personal research priorities [2, 3, 21]. In contrast, methods that require a small group of participants to make a collective decision about priorities, such as the James Lind Alliance approach, have produced ranked lists of priorities [7, 12]. This discrepancy is an important consideration for future priority-setting exercises and suggests that there are a diverse range of research topics of high importance to consumers and carers. This apparent lack of consensus presents a challenge for researchers seeking guidance on where to start in implementing a consumer- and carer-led research agenda. However, within ACACIA, consumers and carers also provided a solution to this dilemma – they suggested that the breadth of priorities reflects the equal importance of all of the topics developed, and research into any of them will address an important gap in the system [14]. Consumers and carers have identified what they need mental health research to address; it is now up to researchers to work in partnership with consumers and carers to implement the agenda.

### Limitations

A potential limitation of this project is that participation in a research process involving researchers and/or mental health professionals can be intimidating for consumer and carer participants and may thereby distort the findings from a priority-setting exercise [8]. However, we consider this to be unlikely in the current research. Forum participants commented on the respectful atmosphere within the forum that contributed to a sense that it was truly consumer- and carer-led, or as one participant commented in an evaluation form “…*acknowledging our capacity to be researchers, not just be researched*”. Participants also remarked in their evaluation forms that the opportunity to discuss their ideas and experiences with other consumers and carers was highly valued.

Due to the large number of items employed, the modest participant sample size in the survey study was not sufficient to conduct inferential statistical comparisons between the consumer, carer and consumer/carer priority ratings. However, the sample size was comparable to previous priority-setting projects with similar methodologies [7, 8, 21]. Additionally, there were a number of characteristics of the sample that may preclude the generalisability of the results, namely (1) the sample self-selected to participate, meaning that the survey may represent the views of those already active in the consumer and carer advocacy spheres, (2) the sample was predominantly female (74%), (3) had an uneven geographical distribution, and (4) 50% of participants fell into the consumer-only category. Recruitment of a diverse sample in future studies may be facilitated by visiting relevant community spaces and engaging with targeted community groups, effectively taking the research to the participants [7, 14]. Drop-out across the survey, potentially due to participant fatigue, reduced the information available about research topics presented late in the survey (Additional file 2). Randomisation of item order may effectively manage this issue in future research.

Although the survey study was developed from the findings of the qualitative study, the research project was not explicitly planned as a two-stage process. The second study to attempt to set clearer priorities and update the agenda using a survey was discussed with the ACACIA Advisory Group on multiple occasions following Study 1, with mixed support [14]. The decision to conduct Study 2 and develop the survey directly from the forum findings was driven by the Advisory Group in 2017, who felt the agenda needed to be updated but the original topics preserved. Developing a survey directly from qualitative work has been a highly successful process in past research conducted by the authors [2]; however, the 4-year gap between studies for the current project and the more specific nature of many of the items compared with previous research may have affected the ease of interpretation of items due to changes in the mental health system in the intervening time.

In both studies, comments indicated that participants found it difficult to prioritise topics as they perceived most to be equally important or as connected to each other. This precluded meaningful analysis of the ranking data and limited the conclusions that could be drawn about clear priorities. Future studies should consider alternative methods to improve consensus; however, it is important that decisions on methods are reached collaboratively with consumers and carers to ensure they are acceptable and avoid reaching misleading conclusions as a result of forced choice.

## Conclusion

Consumers and carers consider many topics important targets for research, suggesting a strong understanding of the Australian mental health system and its failures as discussed in a recent commentary [25]. Consistent with this, in the current study, topics focused on services, particularly the organisation and delivery of care, were seen by consumers and carers as some of the most important on which to focus. People with a lived experience of mental health issues are ideally placed to identify inadequacies in the mental health care system, and have a strong desire for active involvement in addressing these challenges. There is a need to engage in an ongoing research partnership with consumers and carers to ensure that their views are at the forefront of research, and to regularly update research agendas to ensure they are responsive to current consumer and carer priorities. Taking this collaborative approach will move mental health internationally to the forefront of co-designed and co-delivered health systems.

## Additional files

Additional file 1: Forum procedure and prompt list. (DOCX 16 kb)

Additional file 2: Forum consumer, carer and consumer/carer votes on topics and areas for research. (DOCX 20 kb)

Additional file 3: Priority-setting survey. (PDF 798 kb)

## Abbreviations

ACACIA: The Australian Capital Territory Consumer and Carer Mental Health Research Unit
ACT: Australian Capital Territory
ANU: Australian National University
CMHR: Centre for Mental Health Research
NDIS: National Disability Insurance Scheme
