# Supplementary material for: Mental health research priorities in Australia: a consumer and carer agenda
Source: Health Res Policy Syst. 2018 Dec 12;16:119. doi: 10.1186/s12961-018-0395-9 (PMC6292010; doi:10.1186/s12961-018-0395-9)
Supplement: Supplementary file 1 — Forum procedure and prompt list. (DOCX 16 kb) [file 12961_2018_395_MOESM1_ESM.docx]

Additional File 1. Forum procedure and prompt list

| Activity | Group size | Goal | Procedure |
| --- | --- | --- | --- |
| 1 | Small groups (6-8 participants) | To identify research areas important to consumers and carers | Questions asked:  1. “What are the topics/areas/services that you think should be the focus of research within the ACT*?”*.  Included prompts of research topics if needed generated previously [[3](#_ENREF_3)]:   1. Management of mental health issues (including self-management) 2. Treatment 3. Effects of mental health issues (e.g. on carers and families) 4. Causes and risk factors for mental health issues 5. Medication 6. Diagnosis 7. Education and awareness 8. Services 9. Psychological therapies 10. Aspects of everyday life that affect mental health (e.g. diet, sleep) 11. Employment 12. Government policy and funding for mental health 13. Health professional issues 14. Description and characteristics 15. Alternative therapies 16. Research issues (e.g. methods) |
| 2 | Small groups (6-8 participants) | To start refining the ideas generated during the first activity, which allowed consumers and carers to participate in the early stages of thematic analysis [[13](#_ENREF_13)]. | - Facilitators assisted participants to re-examine their earlier ideas and start to formulate possible questions,  - Questions were collated on poster paper and whiteboards. |
| 3 | Large group (All participants) | To enable all participants to prioritise the research topics identified. | Two members of the research team (MB and KG) collated the individual topics generated by the groups in Activity 2 into broad thematic areas by consensus and using an inductive approach;  - Topics organised in the thematic areas were then displayed on poster paper.  - Disagreements were resolved by discussion with the broader research team and Advisory Group members.  - Topics were assigned only to one theme: where a topic related to more than one theme, the primary focus was decided and the topic assigned to the theme that best reflected that focus.  - Themes were refined throughout the coding process to reflect the breadth of topics coded to that area and topics already coded were checked and reassigned as necessary to ensure the coding structure best reflected the data.  - The final list of themes and associated topics was reviewed by the research team for consistency and presented to the forum participants.  - Dot-mocracy priority-setting exercise conducted by allowing participants to place coloured dots next to the priority of their choice. |
