# Supplementary material for: Mental health research priorities in Australia: a consumer and carer agenda
Source: Health Res Policy Syst. 2018 Dec 12;16:119. doi: 10.1186/s12961-018-0395-9 (PMC6292010; doi:10.1186/s12961-018-0395-9)
Supplement: Supplementary file 2 — Forum consumer, carer and consumer/carer votes on topics and areas for research. (DOCX 20 kb) [file 12961_2018_395_MOESM2_ESM.docx]

Additional File 2. Forum consumer, carer, and consumer/carer votes on topics and areas for research

| Topic | Consumer | Carer | Consumer  /carer | TOTAL |
| --- | --- | --- | --- | --- |
| *Services* | | | |  |
| Disconnection of services | 2 | 1 |  | 3 |
| Do the public and private sectors work together? Service user and carer experiences |  |  | 1 | 1 |
| Awareness and role of GPs:  Engagement with carers  Language and communication skills with service users and carers |  |  | 1 | 1 |
| Service pathways:  First access (how do they go about it)  What is the access to information  Benefit of hindsight | 3 | 1 |  | **4*** |
| Trauma-informed care: how is it integrated into service delivery? | 3 | 1 | 3 | **7*** |
| Impact of service delivery on individual:  Service user and carer views on recovery  Service user and carer journey  What works and what doesn’t  What do clinicians think? |  |  |  |  |
| Service user and carer voice integrated into services/policy:  Feedback on effectiveness  How contribution is valued  What are indicators to demonstrate feedback use? |  |  |  |  |
| Monitoring and evaluation: to what extent is it built into program/pre-post-during evaluation from participants? | 2 | 1 |  | 3 |
| Reach – are services reaching the people that need them? | 1 |  |  | 1 |
| How are Partners in Recovery, Personal Helpers and Mentors, support and clinical management working together? |  |  |  |  |
| *Treatment* | | | |  |
| Alternative treatments: what are they?  Holistic approaches  Meditation  Exercise | 1 | 2 |  | 3 |
| ECT:  What information is given?  Does it follow best practice?  What are service users’ experiences? | 2 |  |  | 2 |
| Pet therapy |  |  |  |  |
| Care planning: what makes a good mental health plan? e.g. individualised, including perspectives of service users, carers and clinicians |  | 1 | 1 | 2 |
| Transparency of clinical management |  |  | 1 | 1 |
| Implementation of clinical practice guidelines: why is there a disconnect between evidence and practice? |  |  | 1 | 1 |
| How do current protocols support service user and carer recovery? |  |  |  |  |
| *Medication (2 carer votes for area)* | | | |  |
| How people are supported to come off medication? | 2 |  |  | 2 |
| Gender-specific effects of medication | 1 |  |  | 1 |
| How medications are tailored to the individual? |  |  |  |  |
| Criteria for prescribing | 2 |  |  | 2 |
| How do individuals adapt to change e.g. in medications that impact lifestyle and quality of life |  |  |  |  |
| Is medication what we want?  Side effects  Health impacts  Alternatives  Efficacy  Cost-effectiveness |  |  |  |  |
| *Health professionals* | | | |  |
| Burnout of mental health professionals: impact on service support and delivery |  |  |  |  |
| Getting health professionals working together |  |  |  |  |
| How can service user perspectives be incorporated into psychology training? | 2 |  |  | 2 |
| How is privacy interpreted by health professionals and does it differ from service user and carer interpretation? |  | 1 |  | 1 |
| *Comorbidity/physical health* | | | |  |
| Effects of drug and alcohol use early in life |  | 2 |  | 2 |
| Support when pain is comorbid and how people experience that |  |  |  |  |
| Physical health concerns |  |  |  |  |
| Trial of primary health care nurse with mental health teams |  |  |  |  |
| Evidence base for linking mental illness with alcohol and other drugs |  |  |  |  |
| *Justice system* | | | |  |
| Overrepresentation of mental illness in the justice system | 1 | 1 |  | 2 |
| Discrimination | 1 |  | 1 | 2 |
| *Service user and carer involvement* | | | |  |
| Who is involved? |  |  |  |  |
| How do we expand who is involved? E.g. young people |  |  |  |  |
| How participation works in the ACT (tokenism vs real involvement) | 1 | 1 |  | 2 |
| *Stigma (1 carer vote for area)* | | | |  |
| Stigma by health providers (mental health and others): what do they believe and how does it impact? | 1 |  | 2 | 3 |
| Comorbidities and stigma |  |  |  |  |
| Does the stigma in the mental health system worsen outcomes? | 1 |  |  | 1 |
| Borderline personality disorder and stigma |  |  |  |  |
| Analysis of stigma by disorder |  |  |  |  |
| Stereotype formation |  |  |  |  |
| What changes do people make in their own lives as a result of stigma? | 3 |  |  | 3 |
| *Experiences of care* | | | |  |
| Is care traumatising? | 3 | 2 |  | **5*** |
| How have people who have experienced trauma been cared for? | 1 |  |  | 1 |
| What is helpful in recovery-oriented services?  Are service users being consulted?  Do people know services exist/the pathways into them? |  | 1 |  | 1 |
| *Carers/families and friends (1 service user vote, 1 service user/carer vote)* | | | |  |
| Who are the carers and what are they doing? |  |  |  |  |
| What kind of support would they like? |  |  |  |  |
| Bereavement counselling  Are we offering enough counselling?  Is it timely enough?  Should it be offered in prisons? |  |  |  |  |
| *National Disability Insurance Scheme* | | | |  |
| Impact of NDIS – longitudinal study.  Who is included?  How is it defined?  What are the effects on service users and carers and on service funding? | 1 | 2 | 1 | **4*** |
| People out of NDIS scope: what can be done to reach them? | 2 | 1 | 1 | **4*** |
| *Language and communication* | | | |  |
| How does language include/exclude? | 2 |  |  | 2 |
| Service user perspectives on use of labels | 1 |  |  | 1 |
| What forms of communication work for service users and carers? |  |  | 1 | 1 |
| *Peer-to-peer* | | | |  |
| How to implement internationally recognised models of peer support | 1 |  |  | 1 |
| What are clinician views on peer support? |  |  |  |  |
| Peer-led services | 5 |  | 1 | **6*** |
| What are the gaps? E.g., support groups | 2 | 1 |  | 3 |
| How to recruit and train workers.  What is going where?  Where is it embedded?  How are they being supported? | 3 |  |  | 3 |
| What is a peer? |  |  |  |  |
| Service users’ experiences of peer-to-peer |  |  |  |  |
| *Legislation* | | | |  |
| Capacity for decision making/change in the Mental Health Act and its application: service user and carer experiences of this.   What information is provided about the Act?  What support is provided e.g. legal? | 1 |  |  | 1 |
| To what extent do we follow human rights legislation on mental illness? | 3 |  |  | 3 |
| *Other* | | | |  |
| Impacts on specific age groups (young people, older people) | 2 | 1 | 1 | **4*** |
| Supported accommodation:  What is available versus who needs it?  What levels are available? | 2 | 1 |  | 3 |
| Employment:  Access to relevant opportunities  Understanding of mental health support and services |  | 1 | 1 | 2 |
| Children of people with mental illness/parenting  Attitudes towards parents with mental illness, specifically guardianship orders  How many disclose? Why/why not?  How is status as parents considered in treatment?  How many people have been told not to have children and what is their diagnosis? | 1 |  |  | 1 |
| Smoking cessation for those with mental health issues e.g., after being in a smoke free mental health unit:  What might be helpful for those in this demographic?  Tailored treatments/interventions |  |  |  |  |
| Support in education settings | 1 |  | 1 | 2 |
| How are people from CALD backgrounds experiencing the mental health system? | 1 |  | 1 | 2 |
| Risk factors for mental illness:  Age  Pain/physical health  Trauma  LGBTI | 1 |  |  | 1 |
| Social inclusion:  Models of success  What do we want from it?  Work, employment, education  What makes a good life for service users and carers? |  |  |  |  |
| Recovery and fulfilling potential:  How do current protocols and their implementation support a service user and carer journey to recovery?  What are service users’ experiences of the impact of mental illness on coping, function, day to day activities? | 4 |  | 2 | **6*** |
| Learned helplessness with changes in last 10-20 years with access to support and services – employment, socialising, participation | 3 |  |  | 3 |
| Insurance and recovery: how does the current insurance system affect people living with mental illness? |  |  | 1 | 1 |
| Bullying: how the non-violent therapeutic programs, strategies address and bridge the care of children and youth |  |  | 1 | 1 |
| Bereavement: is there such a thing as carer recovery? |  |  |  |  |
| Suicide: continuous care and support over the lifespan |  |  | 1 | 1 |
