# Supplementary material for: Mental health research priorities in Australia: a consumer and carer agenda
Source: Health Res Policy Syst. 2018 Dec 12;16:119. doi: 10.1186/s12961-018-0395-9 (PMC6292010; doi:10.1186/s12961-018-0395-9)
Supplement: Supplementary file 3 — Priority-setting survey. (PDF 798 kb) [file 12961_2018_395_MOESM3_ESM.pdf]

Consumer and Carer Priorities for Mental Health Research

In November 2013, ACACIA: The ACT Consumer and Carer Mental Health Research Unit held a forum to identify areas for research that are of particular interest to consumers and carers. In the questions to follow, you will be asked to rate the importance of research topics generated during this forum. You will also have the opportunity to suggest new topics.

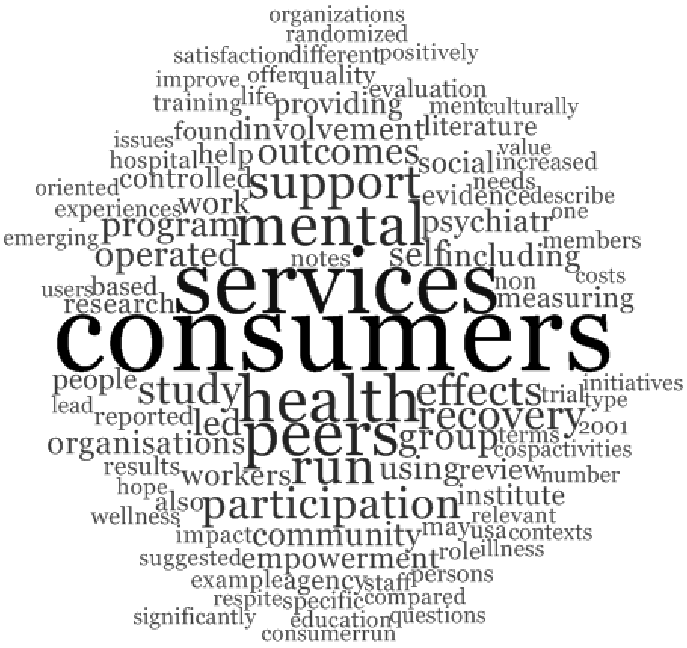

All topics were developed by consumers and carers, and their original wording is used wherever possible. Topics are organised into the following categories:

- Services;
- Treatment;
- Medication;
- Health Professionals;
- Comorbidity & Physical Health;
- Justice System;
- Consumer & Carer Involvement;
- Stigma;
- Experiences of Care;
- Carers, Family & Friends;
- National Disability Insurance Scheme (NDIS);
- Language & Communication;
- Peer to Peer;
- Legislation; and
- Other.

At the end of the survey, you will be asked to rank the topics you selected as most important. All topics rated as "5 - very high priority" will be presented for ranking in order of importance, so that we have a better idea of where to focus our research. We encourage you to think carefully about which topics are of highest priority to you, as it can be challenging to rank a large number of topics.

Your input in this survey will influence the direction of mental health research undertaken by ACACIA. The table below identifies topics previously generated by consumers and carers that are have already been researched or are currently under development.

Topics currently being investigated	Projects underway
• Service pathways- First access, how do they go about it, what is the access to information, benefit of hindsight?<br>• Care planning: what makes a good mental health plan?<br>• Disconnection of services	Finding the Path
• Impacts on specific age groups (young people, older people)	Older people and chronic mental illness
• Monitoring and evaluation - to what extent is it built into program: pre-, post-, during evaluation from participants	Partners in Recovery evaluation
• Consumers' experiences of peer-to-peer<br>• How to recruit and train peer workers.<br>• How are they being supported?<br>• What are clinician views on peer support?	Stay Strong peer worker feasibility study
• Consumer and carer voice integrated into policy	Whose story is it?

Topics under development	Progress
• How to implement internationally recognised models of peer support<br>• Peer-led services<br>• What are the gaps in peer services?<br>• How to recruit and train workers. What is going on where? Where is it embedded? How are they being supported?<br>• What is a peer?<br>• Consumers’ experiences of peer to peer	Two applications for funding from the National Health and Medical Research Council submitted
• Alternative treatments<br>• Impacts on specific age groups	Applications submitted for funding to evaluate a music therapy program for depression in older people

This survey should take approximately 30 minutes to complete. If you pause and exit the survey, you will be able to return to this website and continue completing your responses for up to one week after you begin.

<u>**Definitions of key terms**</u>

Consumers – People who have personal lived experience of mental health issues
Carers - Family members or friends who provide informal support to mental health consumer(s)
Involvement - Consumers and carers *actively* contributing to research, advocacy and services beyond the role of 'subject' or passive participant. In a research context this can include formulation of research questions, design of research protocols, implementation of the research, dissemination of research findings.

<u>**Participant Information Sheet**</u>

Consumer and Carer Priorities for Mental Health Research

Research Team:
This research is being conducted by ACACIA: The ACT Consumer and Carer Mental Health Research Unit. ACACIA is part of the Centre for Mental Health Research at The Australian National University. Our research is conducted in partnership with and for the benefit of mental health consumers and carers. The members of our research team are:
Dr Michelle Banfield, Fellow
Dr Amelia Gulliver, Research Fellow
Alyssa Morse, Research Officer
Owen Forbes, Research Support Officer
Ben Freeman, Research Support Officer

<u>**Description:**</u> In November 2013, ACACIA held its inaugural forum. The purpose of the forum was to identify areas for research that are of particular interest to people with lived experience of mental health issues. ACACIA researchers have been progressing this agenda as described on the previous page and on our webpage (http://cmhr.anu.edu.au/acacia).
The current project consists of an online questionnaire. The purpose of this project is to “check in” with consumers and carers about whether the topics identified in 2013 are still a priority, and give people the chance to suggest new areas that may not be in the list. You will be asked to rate the importance of research topics generated during the 2013 forum, rank the highest priorities and suggest any new topics of importance.

<u>**Participants:**</u> Australian residents over the age of 18 who identify as mental health consumers or carers are invited to complete the survey. A consumer is someone who has personal experience of mental health issues, whether or not they have accessed services. A carer is a family member or friend who provides informal (i.e., unpaid) support to a mental health consumer.

<u>**Use of Data and Feedback:**</u> Survey responses from all participants will be combined to identify the highest priorities. These priorities will be used to shape the ACACIA research agenda. Results will be included in peer-reviewed journal papers and

ACACIA reports. A plain language summary will be made available on the ACACIA website http://cmhr.anu.edu.au/acacia, included in our newsletter and circulated to members of our register.

Project Funding: The project is funded by the Australian Capital Territory Government Health Directorate. This project is part of a wider initiative to involve mental health consumers and carers in research processes.

What's involved?

· **Voluntary Participation & Withdrawal:** Participation in this research is voluntary. You don't have to take part, you don't have to answer all the questions and you can stop the survey at any time by just closing your browser. Responses from partially completed surveys will still be included in our study. If you decide to stop, your responses cannot be deleted as we have no way of identifying individual contributions.

· **What does taking part involve?** In this project you will be asked to complete an online questionnaire. The questions will ask you to rate the priority of a variety of consumer- and carer-generated research topics on a 5-point scale from very low to very high priority. As the purpose is to *prioritise* topics, please think carefully about which ones you rate as "very high". At the end of the survey, you will be asked to rank the topics you rated as very high priority in order of their importance. Throughout the survey you will also have the opportunity to suggest new topics for research if you think anything is missing. New suggestions cannot be included on the ranking page, but you can use the open-ended box at the end of the survey to note if these are your highest priorities.

· **Location and Duration:** You can access the survey from any web-enabled device. We estimate that completion of the questionnaire will take around 30 minutes. We recognise that this is a substantial amount of time but we are keen to ensure that consumer and carer priorities are well covered. You don't have to complete the survey all in one go. You can return to an incomplete survey at any time within a week of starting. Clicking on the survey link on the same device will return to the page where you left off. Your response will automatically submit after one week.

· **Risks:** There is a risk that answering questions about mental health may trigger unpleasant feelings or memories. A link to a list of support services is embedded in the footer of each survey page and will be presented at the end of the survey. If you feel distressed when completing the survey, please contact your preferred support person or one of the services linked from our page.

· **Benefits:** The planned research program will benefit consumers, carers and community stakeholders by ensuring that research conducted by ACACIA is relevant to current priority issues identified by the community. Research into current issues has the potential to influence policy and practice, improving our mental health system.

Confidentiality:

· **Confidentiality:** The survey does not ask for any identifying information. Please do not include identifying information in open responses. Only named research staff will have access to the original survey data. Your data will be kept confidential to the extent permitted by law. No one will be able to identify you by reading our publications or reports.

Privacy Notice:

In collecting your personal information within this research, the ANU must comply with the Privacy Act 1988. The ANU Privacy Policy is available at https://policies.anu.edu.au/ppl/document/ANUP_010007 and it contains information about how a person can:

- Access or seek correction to their personal information;
- Complain about a breach of an Australian Privacy Principle by ANU, and how ANU will handle the complaint.

Participants should be aware that the World Wide Web is an insecure public network that gives rise to a potential risk that a user's transactions are being viewed, intercepted or modified by third parties or that data which the user downloads may contain computer viruses or other defects.

Data Storage:

· **Where:** All data will be accessible only to ACACIA staff on password protected computers. Data collected during the survey will be securely stored on a remote server located in Utah, United States according to ethical guidelines. Data can be uploaded from Australia via a secure portal managed by Qualtrics.

· **How long:** Data associated with this project will be used to shape the ACACIA research agenda until the completion of the current funding agreement in June 2019, after which it will be archived for use in future priority-setting projects should ACACIA continue. The archive is the ANU Data Commons, accessible at https://datacommons.anu.edu.au/DataCommons/ . Archived data will contain no identifying information and may be shared with future staff and students of ACACIA.

Queries and Concerns:

· **Contact Details for More Information:** If you have any queries or concerns regarding this research, please contact Alyssa Morse (e: acacia@anu.edu.au t: +61 2 6125 6167)

Ethics Committee Clearance:

The ethical aspects of this research have been approved by the ANU Human Research Ethics Committee (protocol number 2013/388). If you have any concerns or complaints about how this research has been conducted, please contact:

Ethics Manager
The ANU Human Research Ethics Committee
The Australian National University
Telephone: +61 2 6125 3427
Email: Human.Ethics.Officer@anu.edu.au

I have read and understood the information about this research project. I understand that any information I provide from this point onwards will be included in the research, even if I don't finish the survey.

- Yes
- No

Key Instructions

- **All topics rated as "5 - very high priority" will be selected for further ranking at the end of the survey. We encourage you to think carefully about which topics are of highest priority to you, as it can be challenging to rank a large number of topics.**
- **If moving forward/backward through the survey, only use the "Back" and "Next" buttons at the bottom of each page, not your web browser buttons.**
- **Once you have started the survey, you can close it and return to it within a week on the same device.**
- **Throughout the survey you will have the opportunity to suggest new topics if you think anything is missing. New suggestions cannot be included on the final ranking page, but you can use the open-ended box at the end of the survey to note if these are your highest priorities.**

Which of the following roles do you identify with? More Information A consumer is someone who has personal experience of mental health issues, whether or not they have accessed services. A carer is a family member or friend who provides informal (i.e., unpaid) support to a mental health consumer.

- ○ Consumer
- ○ Carer
- ○ Consumer and carer
- ○ None of the above

If "None of the above", then:

Thank you for your interest in this study. At this time we are only seeking participation from people over the age of 18 who identify as mental health consumers or carers. If you are interested in joining our register or participating in future ACACIA studies, you can find our website here.

Else, continue the survey

Demographic Information

What is your age? (In years)

What is your gender?

- ○ Male
- ○ Female
- ○ Other
- ○ Prefer not to say

Where do you live?

ACT
NSW
VIC
QLD
NT
WA
TAS

Services

All topics rated as "5 - very high priority" will be selected for further ranking at the end of the survey. We encourage you to think carefully about which topics are of highest priority to you, as it can be challenging to rank a large number of topics.

	Very low priority 1	2	3	4	Very high priority 5
Do the public and private sectors work together? Consumer and carer experiences	○	○	○	○	○
Awareness and role of General Practitioners (GPs) - e.g., engagement with carers, language and communication skills with consumers and carers	○	○	○	○	○
Trauma informed care - Why is it important, and how is it integrated into service delivery?	○	○	○	○	○
Impact of service delivery on consumers and carers - What contributes to recovery?	○	○	○	○	○
Consumer and carer journey through service pathways - What works and what doesn't? What do clinicians think?	○	○	○	○	○
How is the consumer and carer voice integrated into policy and services? How are their contributions valued, and what indicators exist to demonstrate how their voice is used?	○	○	○	○	○
Reach – Are services reaching the people that need them?	○	○	○	○	○
How are Partners in Recovery (PiR), Personal Helpers and Mentors (PHaMs), support and clinical management working together?	○	○	○	○	○

Please add any additional comments or suggested topics relevant to this theme below.

Treatment

All topics rated as "5 - very high priority" will be selected for further ranking at the end of the survey. We encourage you to think carefully about which topics are of highest priority to you, as it can be challenging to rank a large number of topics.

	Very low priority 1	2	3	4	Very high priority 5
Alternative treatments - What are they, and how can they contribute to recovery? Holistic approaches, meditation, exercise	○	○	○	○	○
Electroconvulsive therapy (ECT) - What information is given, does it follow best practice, what are consumers' experiences?	○	○	○	○	○
Pet therapy	○	○	○	○	○
Care planning - What makes a good mental health plan? (e.g. individualised, including perspectives of consumers, carers and clinicians)	○	○	○	○	○
Transparency of clinical management - How does it respond and interact with consumers and carers?	○	○	○	○	○
Translation of clinical frameworks and guidelines into practice - Why is there a disconnect?	○	○	○	○	○
How do current protocols support consumer and carer journeys to recovery?	○	○	○	○	○

Please add any additional comments or suggested topics relevant to this theme below.

Medication

All topics rated as "5 - very high priority" will be selected for further ranking at the end of the survey. We encourage you to think carefully about which topics are of highest priority to you, as it can be challenging to rank a large number of topics.

	Very low priority 1	2	3	4	Very high priority 5
What are the experiences of and needs of people coming off medication? How are they being supported?	◯	◯	◯	◯	◯
Gender specific effects of medication	◯	◯	◯	◯	◯
How can medications be tailored to the individual?	◯	◯	◯	◯	◯
Criteria for prescribing medications	◯	◯	◯	◯	◯
How do individuals adapt to changes in medication that impact lifestyle and quality of life?	◯	◯	◯	◯	◯
Is medication what we want? Side effects, health impacts, alternatives, efficacy, cost-effectiveness	◯	◯	◯	◯	◯

Please add any additional comments or suggested topics relevant to this theme below.

Health Professionals

All topics rated as "5 - very high priority" will be selected for further ranking at the end of the survey. We encourage you to think carefully about which topics are of highest priority to you, as it can be challenging to rank a large number of topics.

	Very low priority 1	2	3	4	Very high priority 5
Exhaustion & burnout of mental health professionals - Impact on service support and delivery	○	○	○	○	○
How can we get mental health and other health professionals to work together more efficiently?	○	○	○	○	○
Training of psychologists - How can consumer perspectives be incorporated?	○	○	○	○	○
How is 'privacy' interpreted by health professionals, and does it differ from consumer and carer interpretations?	○	○	○	○	○
How mental health-aware are General Practitioners (GPs)?	○	○	○	○	○
What is the role of a General Practioner (GP) - perceived and actual) - as part of the therapeutic alliance in care of mental health consumers?	○	○	○	○	○

Please add any additional comments or suggested topics relevant to this theme below.

Comorbidity & Physical Health

All topics rated as "5 - very high priority" will be selected for further ranking at the end of the survey. We encourage you to think carefully about which topics are of highest priority to you, as it can be challenging to rank a large number of topics.

	Very low priority 1	2	3	4	Very high priority 5
Effects of drug and alcohol use early in life	◯	◯	◯	◯	◯
What support is available when pain is a comorbid condition? How are people experiencing that?	◯	◯	◯	◯	◯
Where do physical health concerns fit into health services when you have mental health problems as a main focus?	◯	◯	◯	◯	◯
Care coordination between mental health and physical health	◯	◯	◯	◯	◯
Trial of primary health care nurse within mental health teams – Does it improve physical health outcomes?	◯	◯	◯	◯	◯
What is the evidence base linking mental illness with alcohol and other drugs?	◯	◯	◯	◯	◯

Please add any additional comments or suggested topics relevant to this theme below.

Justice System

All topics rated as "5 - very high priority" will be selected for further ranking at the end of the survey. We encourage you to think carefully about which topics are of highest priority to you, as it can be challenging to rank a large number of topics.

	Very low priority 1	2	3	4	Very high priority 5
Over-representation of mental illness in the justice system	◯	◯	◯	◯	◯
Discrimination	◯	◯	◯	◯	◯

Please add any additional comments or suggested topics relevant to this theme below.

Consumer & Carer Involvement

All topics rated as "5 - very high priority" will be selected for further ranking at the end of the survey. We encourage you to think carefully about which topics are of highest priority to you, as it can be challenging to rank a large number of topics.

	Very low priority 1	2	3	4	Very high priority 5
Consumers & Carers - Who is involved?	◯	◯	◯	◯	◯
How do we expand who is involved? (e.g. young people)	◯	◯	◯	◯	◯
How participation works in practice (tokenism vs. real involvement)	◯	◯	◯	◯	◯

Please add any additional comments or suggested topics relevant to this theme below.

Stigma

Stigma is when someone is seen in a negative way because of their mental health issues. When a person is labelled by their mental illness they are no longer seen as an individual but as part of a stereotyped group. Negative attitudes and beliefs toward this group create prejudice which can lead to negative actions and discrimination.

All topics rated as "5 - very high priority" will be selected for further ranking at the end of the survey. We encourage you to think carefully about which topics are of highest priority to you, as it can be challenging to rank a large number of topics.

	Very low priority 1	2	3	4	Very high priority 5
Stigma by health providers (mental health and others) - What do they believe and how does it impact?	○	○	○	○	○
Comorbidities and stigma	○	○	○	○	○
Does the stigma in the mental health system worsen outcomes?	○	○	○	○	○
Stigma around Borderline Personality Disorder	○	○	○	○	○
Analysis of stigma by disorder	○	○	○	○	○
Stereotype formation	○	○	○	○	○
What changes do people make in their own lives as a result of stigma?	○	○	○	○	○
Stigma as a barrier to consumer involvement	○	○	○	○	○

Please add any additional comments or suggested topics relevant to this theme below.

Experiences of Care

All topics rated as "5 - very high priority" will be selected for further ranking at the end of the survey. We encourage you to think carefully about which topics are of highest priority to you, as it can be challenging to rank a large number of topics.

	Very low priority 1	2	3	4	Very high priority 5
Is care traumatising?	○	○	○	○	○
How have people who have experienced trauma been cared for?	○	○	○	○	○
What is helpful in recovery-oriented services?	○	○	○	○	○
Are consumers being consulted about their experiences of care?	○	○	○	○	○

Please add any additional comments or suggested topics relevant to this theme below.

Carers, Family and Friends

All topics rated as "5 - very high priority" will be selected for further ranking at the end of the survey. We encourage you to think carefully about which topics are of highest priority to you, as it can be challenging to rank a large number of topics.

	Very low priority 1	2	3	4	Very high priority 5
Who are the carers and what are they doing?	○	○	○	○	○
What kind of support would carers like?	○	○	○	○	○
Carers & bereavement – Are we offering enough counselling? Is it timely enough? Should it be offered in prisons?	○	○	○	○	○
What is the effect of caring?	○	○	○	○	○
Is there such a thing as carer recovery?	○	○	○	○	○

Please add any additional comments or suggested topics relevant to this theme below.

National Disability Insurance Scheme (NDIS)

All topics rated as "5 - very high priority" will be selected for further ranking at the end of the survey. We encourage you to think carefully about which topics are of highest priority to you, as it can be challenging to rank a large number of topics.

	Very low priority 1	2	3	4	Very high priority 5
What programs/supports can be devised for reaching individuals that are outside of NDIS scope?	◯	◯	◯	◯	◯
How many people with mental illness/disability are eligible for NDIS support?	◯	◯	◯	◯	◯
How is psychosocial disability defined in the NDIS, and how will it impact consumers and carers in Australia?	◯	◯	◯	◯	◯

Please add any additional comments or suggested topics relevant to this theme below.

Language and Communication

All topics rated as "5 - very high priority" will be selected for further ranking at the end of the survey. We encourage you to think carefully about which topics are of highest priority to you, as it can be challenging to rank a large number of topics.

	Very low priority 1	2	3	4	Very high priority 5
How does the use of language include/exclude individuals?	○	○	○	○	○
Consumer perspectives on use of labels - Which terms are useful/helpful, which are not?	○	○	○	○	○
What forms of communication work for consumers and carers? (e.g. older people - less technology familiarity; younger people - social media, smart phones)	○	○	○	○	○
What sources of information do consumers and carers have faith in?	○	○	○	○	○

Please add any additional comments or suggested topics relevant to this theme below

Peer to Peer

All topics rated as "5 - very high priority" will be selected for further ranking at the end of the survey. We encourage you to think carefully about which topics are of highest priority to you, as it can be challenging to rank a large number of topics.

	Very low priority 1	2	3	4	Very high priority 5
How to implement internationally recognised models of peer support in Australia	○	○	○	○	○
What are clinician views on peer support?	○	○	○	○	○
Peer-led services - What are the gaps? (e.g. support groups)	○	○	○	○	○
How to recruit and train peer workers - What is going on, and where? Where is it embedded? How are they being supported?	○	○	○	○	○
What is a peer?	○	○	○	○	○
Consumers' experiences of peer to peer services	○	○	○	○	○
Peer support in public mental health system	○	○	○	○	○

Please add any additional comments or suggested topics relevant to this theme below.

Legislation

All topics rated as "5 - very high priority" will be selected for further ranking at the end of the survey. We encourage you to think carefully about which topics are of highest priority to you, as it can be challenging to rank a large number of topics.

	Very low priority 1	2	3	4	Very high priority 5
Capacity for decision making/change in legislation and its application; consumer and carer experiences of this. What information is provided about legislation? What support is provided e.g. legal?	◯	◯	◯	◯	◯
To what extent do we follow human rights legislation on mental illness?	◯	◯	◯	◯	◯

Please add any additional comments or suggested topics relevant to this theme below.

Block 16

Ungrouped/Other

All topics rated as "5 - very high priority" will be selected for further ranking at the end of the survey. We encourage you to think carefully about which topics are of highest priority to you, as it can be challenging to rank a large number of topics.

	Very low priority 1	2	3	4	Very high priority 5
Accommodation	○	○	○	○	○
Employment	○	○	○	○	○
Children of people with mental illness	○	○	○	○	○
Smoking cessation	○	○	○	○	○
Support in education settings	○	○	○	○	○
Mental health in culturally and linguistically diverse populations	○	○	○	○	○
Culturally and linguistically diverse perspectives within mainstream mental health system	○	○	○	○	○
Risk factors for mental illness	○	○	○	○	○
Social inclusion	○	○	○	○	○
Recovery and fulfilling potential	○	○	○	○	○
Learned helplessness (experience with services)	○	○	○	○	○
Mental health in LGBTIQ+ populations	○	○	○	○	○
Insurance	○	○	○	○	○
Bullying	○	○	○	○	○
Bereavement	○	○	○	○	○
Suicide: continuous care and support	○	○	○	○	○

Please add any additional comments or suggested topics relevant to this theme below.

From the topics you ranked as "5- very high priority", please **drag and drop to rank topics in order of highest to lowest priority.**

On a touch screen device you will need to use two fingers to scroll up or down on this page. Any subjects you have suggested in previous text boxes will not be included for ranking. Please list any suggested topics you feel are of particular importance in the text box on the next page.

Please provide any additional comments or suggested topics which you feel were not covered in the previous sections

Thank you for your time and assistance.

If you experience any distress following this survey, a list of services and support contacts is available here.

Any publications or updates regarding this survey will be available from the ACACIA website.

Hitting the "Next" button below will submit your responses.

If you are feeling distressed at any time, a list of support contacts and services is available here

Powered by Qualtrics
